# Diagnostic Accuracy Study of a Novel Blood-Based Assay for Identification of Tuberculosis in People Living with HIV

**DOI:** 10.1128/JCM.01643-20

**Published:** 2021-02-18

**Authors:** Erik Södersten, Stefano Ongarello, Anna Mantsoki, Romain Wyss, David H. Persing, Sara Banderby, Linda Strömqvist Meuzelaar, Jacqueline Prieto, Devasena Gnanashanmugam, Purvesh Khatri, Samuel G. Schumacher, Claudia M. Denkinger

**Affiliations:** a Cepheid AB, Solna, Sweden; b FIND, Geneva, Switzerland; c Cepheid, Sunnyvale, California, USA; d Institute for Immunity, Transplantation and Infection, Stanford University School of Medicine, Stanford, California, USA; e Department of Medicine, Division of Biomedical Informatics Research, Stanford University School of Medicine, Stanford, California, USA; f Division of Tropical Medicine, Center for Infectious Diseases, Heidelberg University Hospital, Heidelberg, Germany; UNC School of Medicine

**Keywords:** 3-gene, Cepheid, gene signature, Xpert, diagnostics, host response, human immunodeficiency virus, nonsputum, rapid tests, tuberculosis

## Abstract

A nonsputum triage test to rule out tuberculosis (TB) disease is a WHO high-priority diagnostic, and a combinatory score based on a 3-gene host signature has shown promise in discriminating TB from other illnesses. We evaluated the accuracy of an early-prototype cartridge assay (“Xpert MTB Host Response” or Xpert-MTB-HR-Prototype) of this 3-gene signature on biobanked blood samples from people living with HIV (PLHIV) against a comprehensive microbiological reference standard (CMRS) and against Xpert MTB/RIF on the first sputum sample alone. We depict results based on performance targets set by the WHO in comparison with a laboratory-based C-reactive protein (CRP) assay. Of 201 patients included, 67 were culture positive for Mycobacterium tuberculosis. The areas under the concentration-time curve (AUCs) for Xpert-MTB-HR-Prototype were 0.89 (confidence interval [CI], 0.83 to 0.94) against the CMRS and 0.94 (CI, 0.89 to 0.98) against Xpert MTB/RIF. Considering Xpert-MTB-HR-Prototype as a triage test (at the nearest upper value of sensitivity to 90%), specificities were 55.8% (CI, 47.2 to 64.1%) compared to the CMRS and 85.9% (CI, 79.3 to 90.7%) compared to Xpert MTB/RIF as confirmatory tests. Considering Xpert-MTB-HR-Prototype as a stand-alone diagnostic test, at a specificity near 95%, the test achieved a sensitivity of 65.7% (CI, 53.7 to 75.9%), while the CRP assay achieved a sensitivity of only 13.6% (CI, 7.3 to 23.4%). In this first accuracy study of a prototype blood-based host marker assay, we show the possible value of the assay for triage and diagnosis in PLHIV.

## Abstract (precis)

A nonsputum triage test to rule out tuberculosis (TB) disease is a WHO high-priority diagnostic, and a combinatory score based on a 3-gene host signature has shown promise in discriminating TB from other illnesses. We evaluated the accuracy of an early-prototype cartridge assay (“Xpert MTB Host Response” or Xpert-MTB-HR-Prototype) of this 3-gene signature on biobanked blood samples from people living with HIV (PLHIV) against a comprehensive microbiological reference standard (CMRS) and against Xpert MTB/RIF on the first sputum sample alone.

## INTRODUCTION

In 2018, 10 million people developed tuberculosis (TB) disease, and 1.5 million people lost their lives to the illness (1). According to the World Health Organization’s (WHO’s) End TB Strategy, improved diagnostic tests are required as a means to stop the global tuberculosis epidemic by 2035. The prioritized diagnostic tests to be developed were defined in a target-setting exercise in 2014 (1). One of the highest-priority target product profiles (TPPs) is a nonsputum triage test to rule out disease with a minimum of 90% sensitivity and 70% specificity (optimally, 95% sensitivity and 80% specificity) to be used by first-contact providers to identify patients who need further confirmatory testing (1). Currently, no triage test can be done to meet these targets from a nonsputum sample. Chest X ray is widely used as a screening test but is limited by the infrastructure and instrumentation needs and its lack of specificity for TB (2). An optimal novel test should be performable on a readily accessible patient sample such as blood or urine and have limited infrastructure needs (1, 3). The detection of C-reactive protein (CRP) in lateral-flow tests is currently the only test that meets operational characteristics of a triage test, but performance evaluations show limited specificity and have been mostly restricted to populations with a high HIV prevalence (4).

However, novel tests show promise. In 2016, Sweeney et al. reported a multicohort study based on 14 publicly available microarray data sets obtained from whole-blood samples from patients with symptoms compatible with TB disease. The study suggested that a combinatory score (TB score) based on blood mRNA levels of only three differentially expressed genes (*GBP5*, *DUSP3*, and *KLF2*) can discriminate between TB disease and other diseases (5). The derived 3-gene set showed a statistically significant enrichment in M1 macrophages (*P* < 0.05), which are part of the interferon gamma-mediated host response to active tuberculosis.

Cepheid (Sunnyvale, CA, USA) has developed an early-prototype GeneXpert PCR test, Xpert-MTB-HR-Prototype, that quantifies relative mRNA levels of the 3-gene signature in a patient whole-blood sample. The Xpert-MTB-HR-Prototype test is intended for direct analysis of fingerprick blood but can also be utilized retrospectively with blood samples that have been conserved in PAXgene buffer. This study is a first feasibility study to evaluate the performance of Cepheid’s novel assay prototype on preserved blood samples from people living with HIV (PLHIV). PLHIV are a particularly vulnerable patient group with high proportions of extrapulmonary TB or cases of pulmonary TB that are unable to produce sputum (6).

Based on the data provided by Sweeney et al., both triage and stand-alone diagnosis use cases for the signature can be envisioned. Here, we assess the performance of the novel blood-based 3-gene signature cartridge prototype as a non-sputum-based triage test or a stand-alone diagnostic test for TB disease against a microbiological reference standard (culture and Xpert MTB/RIF) and separately, which is more likely to occur when implemented in limited-resource settings and thus is recommended by the WHO, against Xpert MTB/RIF alone.

## MATERIALS AND METHODS

### Study design and participants.

We assessed biobanked blood samples collected as part of the FIND biobanking effort (https://www.finddx.org/specimen-bank/) from inpatients (>18 years of age) living with HIV, independent of CD4 count, in a South African district hospital and a Peruvian referral hospital between February 2016 and August 2017. Participants were adults with TB symptoms who were able to produce sputum. Participants with presumed extrapulmonary disease only were excluded to assess test performance on a well-characterized clinical condition, pulmonary tuberculosis, against reference standards that allows the correct categorization in most cases. The samples were preserved in temperature-controlled freezers at −80°C from collection until testing.

The study was a nested case-control study. The criterion for the selection of a sample was the availability of PAXgene tubes (Becton, Dickinson, Franklin Lakes, NJ, USA), with which a comprehensive work-up was performed to identify TB or rule it out. The participants selected for this study (201 total) were representative of the larger cohort (259 participants with HIV) with respect to the distribution of TB cases and smear status among the TB cases. Given the exploratory nature of the study, no sample size calculations were performed.

All study-related activities were approved by the Human Research Ethics Committee (HREC) of the University of Cape Town (UCT) and the Universidad Peruana Cayetano Heredia (UPCH). Written informed consent was obtained from participants according to the study protocol. Study participation did not affect the standard of care. Reporting followed STARD guidelines (7).

### Index test.

Retrospective testing of PAXgene-conserved blood samples with the index test was performed by Cepheid in January 2019. The index test, early Xpert-MTB-HR-Prototype, evaluates the mRNA levels of three differentially expressed genes (*GBP5*, *DUSP3*, and *KLF2*) (5). For testing, 1 aliquot per patient of 380 μl thawed PAXgene-conserved blood was centrifuged. The supernatant was gently decanted, and any excess liquid from the side of the tube was removed. The pellet was resuspended with a lysis buffer (Cepheid) and vortexed. The lysate was then transferred to the Xpert-MTB-HR-Prototype cartridge and subsequently analyzed on a GeneXpert instrument. A TB score was computed from threshold cycle (*C_T_*) values obtained by the GeneXpert analysis according to the following formula suggested by Sweeney et al. (5): TB score = (*C_T GBP5_* +*C_T DUSP3_*)/2 − *C_T KLF2_*. The calculated score appears different than the one calculated using microarray data. With microarrays, genes that are upregulated receive a higher intensity value. Since the Xpert assay uses reverse transcriptase PCR (RT-PCR) to measure the expression of a gene, expression is measured as a *C_T_* value, which represents the number of cycles for amplifying the corresponding mRNA. When using RT-PCR, a gene expressed at a higher level requires a lower number of cycles for amplification.

### Comparator test.

Serum CRP levels were measured using the Multigent CRP Vario assay on the Abbott Architect C8000 platform at Quest Diagnostics (8). The Multigent CRP Vario assay is a latex immunoassay. An antigen-antibody reaction occurs between CRP in the sample and anti-CRP antibody on latex particles. The resulting agglutination is detected as an absorbance change (572 nm), with the rate of the change being proportional to the quantity of CRP in the sample. The actual concentration is then determined by interpolation from a calibration curve prepared from calibrators of known concentrations. For testing, in brief, serum was thawed and then processed and interpreted according to the manufacturer’s protocol.

### Reference standard.

Fresh sputum, blood, and urine specimens were processed using standardized protocols in centralized accredited laboratories of the South African National Health Laboratory Service and the UPCH. The testing flow for the samples tested are shown in Fig. S1 in the supplemental material. Standard testing was performed on all available sputum specimens and included Xpert MTB/RIF (Cepheid, Sunnyvale, CA, USA) (testing predated the rollout of Xpert MTB/RIF Ultra), smear fluorescence microscopy after auramine staining, MGIT liquid culture (Becton, Dickinson, Franklin Lakes, NJ, USA), and solid culture on Lowenstein-Jensen (LJ) medium. The presence of the Mycobacterium tuberculosis complex in solid and liquid cultures was confirmed with MPT64 antigen detection and/or MTBDRplus line probe assays (Hain Lifesciences, Nehren, Germany). Blood cultures from all participants were done in Bactec Myco/F lytic culture vials (Becton, Dickinson, Franklin Lakes, NJ, USA), and WHO-prequalified *in vitro* diagnostic tests were used for HIV testing (rapid diagnostic tests) and CD4 cell counting (flow cytometry). For urinary Xpert MTB/RIF testing, 20 to 40 ml urine was centrifuged; following the removal of the supernatant, the pellet was resuspended in the residual urine volume, and 0.75 ml was tested using Xpert. The operators of the index were not blind to the results of the reference standard.

### Case definitions.

Participants were assigned to diagnostic categories using a combination of laboratory and clinical findings (Table S1). This was done by clinical investigators prior to performing the index test. Using a comprehensive microbiological reference standard (CMRS), “definite TB” included participants with microbiologically confirmed M. tuberculosis (any culture from sputum or blood or any Xpert MTB/RIF assay from sputum or urine positive for M. tuberculosis during admission). “Non-TB” included participants with all microscopy tests, cultures, and Xpert MTB/RIF tests negative for M. tuberculosis (and at least one noncontaminated culture result) who were not started on anti-TB treatment and were alive and improved at 2 months of follow-up. The non-TB group was further subdivided into patients with and those without evidence of latent TB infection (LTBI) based on testing using commercial interferon gamma release assays (IGRAs). “Possible TB” included any patient not meeting the definite TB or non-TB classification who was started on TB treatment. The “subclinical TB” group was defined as patients presenting with clinical symptoms suggestive of TB and negative microbiological work-up at the time of the first presentation but positive microbiological work-up at follow-up 2 months after the first presentation. In order to confine the analysis of a first prototype assay using the best-characterized patient cohort, participants with subclinical TB and possible TB were excluded from the primary analysis and reported separately (*n* = 5).

In a separate analysis, definite TB was defined by Xpert MTB/RIF positivity on the first sputum sample only (“Xpert MTB/RIF-only” reference standard). This reference standard was used to most closely mimic what is likely to be the confirmatory test in resource-limited settings (as recommended in the TPP by the WHO and based on the fact that sputum culture has seen limited penetration in resource-limited settings due to infrastructure needs and slow turnaround times) (1). Non-TB participants in this analysis were all participants who were Xpert MTB/RIF test negative on the first sputum sample.

### Analysis.

All samples had complete data on reference standard testing. The diagnostic performance of the obtained TB score was evaluated by ROC (receiver operating characteristic) curve analysis using the diagnostic categories definite TB and non-TB as binary reference standards for the classifier. In the primary analysis, the categories definite TB and non-TB were defined by the CMRS. In a secondary analysis, the categories definite TB and non-TB were defined based on the Xpert MTB/RIF result on the first sputum sample as a reference standard.

The following three separate analyses were performed for each of the two different reference standards: (i) sensitivity and specificity were calculated at the threshold value that maximized the Youden index; (ii) to evaluate if the test fulfills the WHO requirements for a triage test, specificity was calculated at the closest threshold value corresponding to 90% sensitivity (minimal target); and (iii) the same analysis as the second one was performed for a sensitivity of 95% (optimal target).

In addition, we explored if the Xpert-MTB-HR-Prototype performance would meet the TPP for a non-sputum-based stand-alone diagnostic test where specificity is optimized (1). While the specificity was set by the WHO at 98% in the TPP, we decided to set it at 95% in our analysis as we recognize the limitations of the reference standard, particularly in PLHIV. We restricted this analysis to a comparison against a CMRS only. Confidence intervals (CIs) reported were calculated based on the Wilson method.

In the statistical analysis plan, the following subgroups were prespecified for subgroup analysis: smear status, CD4 count (categorized as ≤200 and >200 cells/mm^3^), and number of symptoms (categorized as ≤3 or >3).

Symptoms were specified as cough, fever, night sweats, and weight loss for at least 2 weeks. Another prespecified analysis included an assessment of specificity in the two subgroups non-TB without LTBI and non-TB with LTBI based on IGRA results. Indeterminate results were not included in the analysis but reported separately.

## RESULTS

### Performance evaluation of the Xpert-MTB-HR-Prototype test against the comprehensive microbiological reference standard.

A total of 201 samples from South African and Peruvian cohorts of patients diagnosed with HIV were included in the study. Of the 201 patients, 67 (33.3%) were diagnosed with TB, with 23 (34.3%) being smear negative/culture positive and 44 (65.7%) being smear positive/culture positive. Five patients were defined as having subclinical TB, and 129 (64.2%) were categorized as non-TB patients (46 LTBI and 83 non-LTBI). No patient with possible TB was included. Xpert MTB/RIF on the first sputum sample was able to identify 53 patients (79.1%; 43/44 smear positive [97.7%] and 10/23 smear negative [43.5%]), while Xpert MTB/RIF from all sputum samples detected 57 patients (85%).

The patient characteristics are reported in Table 1. The median age was 36 years, with 65% of the participants being female. All patients had confirmed HIV infection, and for all but 10, the CD4 count was available, with a median CD4 count of 375 cells/mm^3^ and 58 patients with a CD4 count of <200 cells/mm^3^. All but 20 patients had more than three symptoms suggestive of TB.

**TABLE 1:** Patient characteristics

Patient characteristic^*a*^	Value (*n* = 201)
Median age (yrs) (IQR)	36 (31–43)

No. of patients of sex (%)	
Female	130 (65.0)
Male	71 (35.0)

No. of patients with TB status (%)	
TB^+^ (% of culture positive)	
S^−^ C^+^	23 (34.3)
S^+^ C^+^	44 (65.7)
TB^−^ (% of all)	134 (66.7)

CD4 count (cells/mm^3^)	
No. of patients with CD4 count of <200 (% with CD4 count)	58 (30.0)
No. of patients with CD4 count of ≥200 (% with CD4 count)	133 (70.0)
No. of patients with unknown CD4 count (% of total)	10 (5.0)
Median CD4 count (IQR)	375 (154–596)

No. of patients with history of BCG vaccination (% of total)	
Positive	177 (88.1)
Negative	4 (2.0)
Unknown and scar indeterminate	20 (10.0)

No. of patients with previous history of TB (% of total)	
Positive	128 (63.7)
Negative	73 (36.3)

No. of patients with QuantiFERON result (%)	
Positive (% with result)	78 (38.8)
Negative (% with result)	95 (47.3)
Indeterminate (% with result)	26 (12.9)
Not obtained (% of total)	2 (1.0)

No. of patients at site of study (%)	
South Africa	195 (97.0)
Peru	6 (3.0)

a Abbreviations: TB^+^, tuberculosis positive; TB^−^, tuberculosis negative; S^−^, sputum negative; S^+^, sputum positive; C^−^, culture negative; C^+^, culture positive; BCG, Mycobacterium bovis BCG.

**FIG 1:**
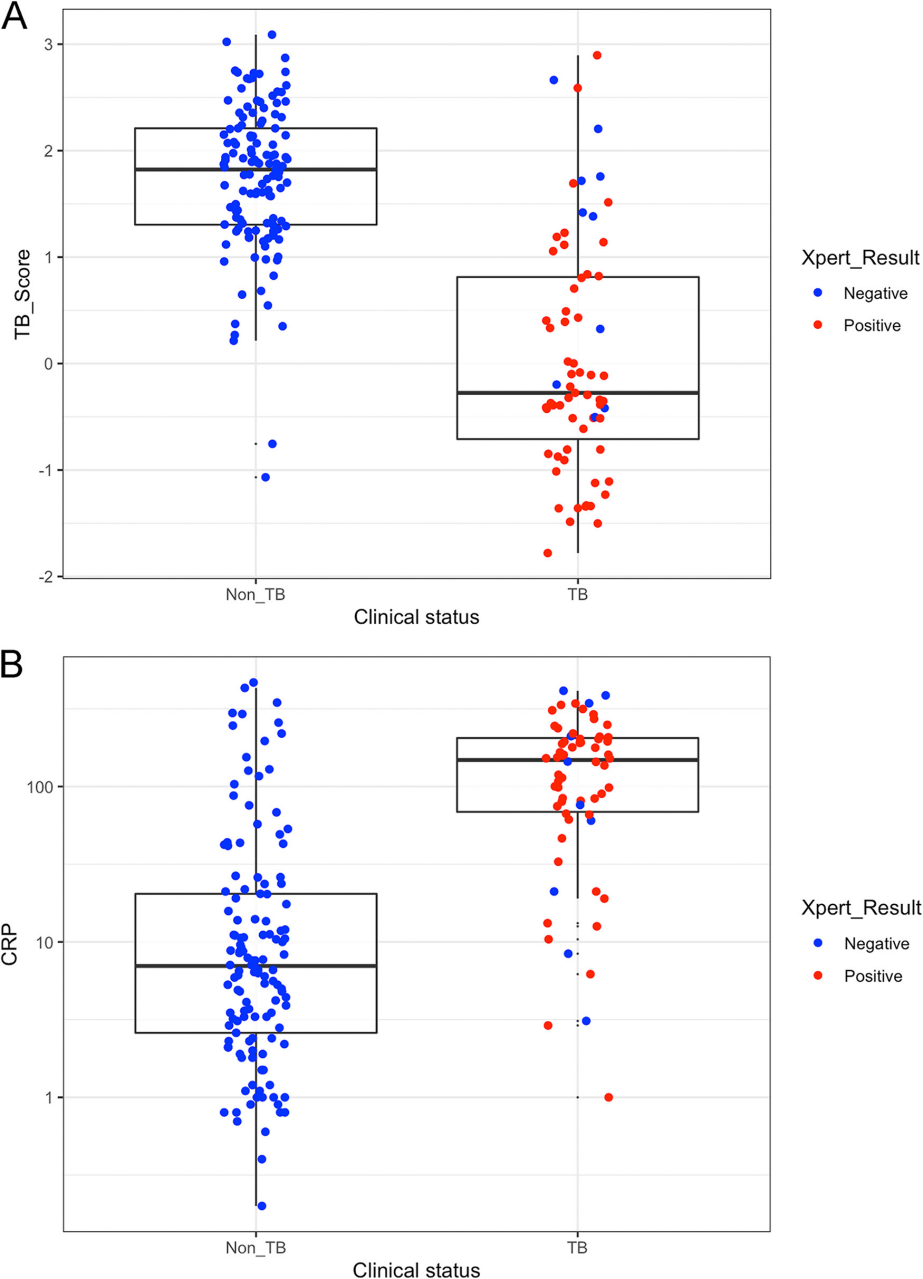
Values for Xpert-MTB-HR-Prototype (A) and the laboratory-based CRP test (B) against a comprehensive microbiological reference standard subdivided by the Xpert MTB/RIF result on the first sputum sample.

The TB score values across participants with and without TB as defined by the CMRS, further subdivided by the Xpert MTB/RIF result on the first sputum sample, are depicted in Fig. 1A. Figure 1B shows the same for CRP. Interestingly, 8 patients identified by the CMRS with paucibacillary diseases were negative by both the TB score and CRP assay.

**FIG 2:**
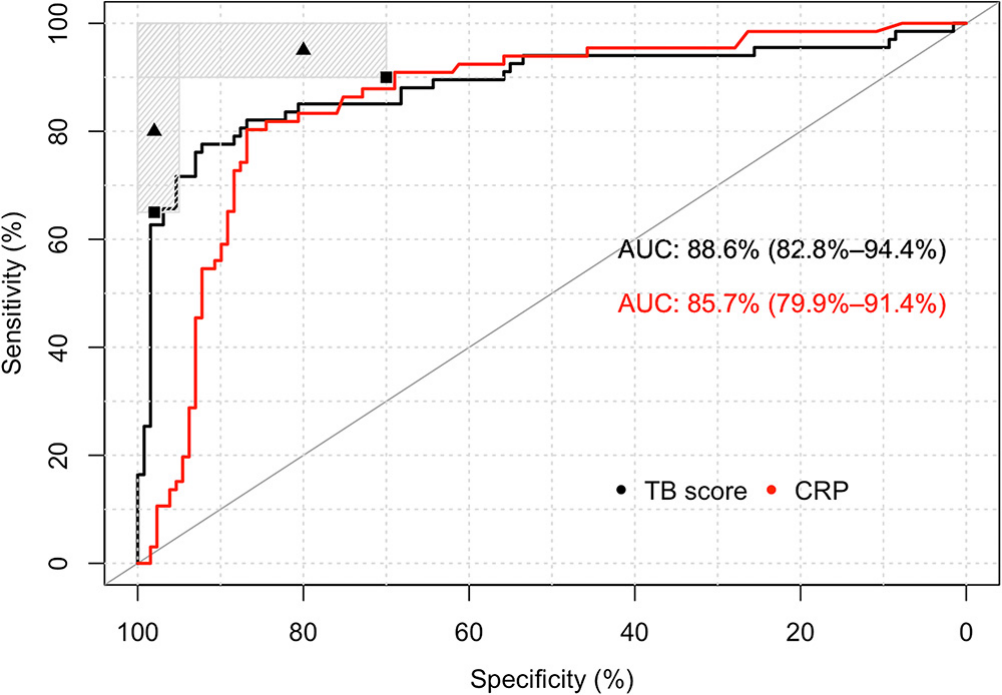
ROC curve for the Xpert-MTB-HR-Prototype test and the laboratory-based CRP test against a comprehensive microbiological reference standard. The shaded regions represent areas with sensitivity and specificity combinations that meet at least the minimal target of one of the TPPs (triage or nonsputum diagnostic). The triangles represent the optimal targets, and the squares represent the minimal targets.

The performance of the Xpert-MTB-HR-Prototype test was first evaluated against the CMRS, which yielded an area under the concentration-time curve (AUC) of 0.89 (95% confidence interval [CI], 0.83 to 0.94) (Fig. 2). As a comparison, the CRP assay yielded an AUC of 0.86 (CI, 0.80 to 0.91) (Fig. 2). Using DeLong’s test, the areas under the ROC curves (AUROCs) for the Xpert-MTB-HR-Prototype and CRP assays were not statistically different (*P* > 0.3) (9). Optimal sensitivity and specificity were obtained for Xpert-MTB-HR-Prototype when the Youden index was maximized. At this TB score, the cut-point sensitivity was 77.6% (CI, 66.3 to 85.9%), and the specificity was 92.2% (CI, 86.3 to 95.7%) (Fig. 2). In comparison, the CRP value at the Youden index was 60.3 mg/liter, which translated into 80.3% sensitivity (CI, 69.1 to 88.1%) and 86.8% specificity (CI, 79.9 to 91.6%) (Fig. 2).

Considering Xpert-MTB-HR-Prototype as a triage test at a fixed value of sensitivity near 90% (91.0% nearest upper value [CI, 81.8 to 95.8%]), the corresponding specificity was 55.8% (CI, 47.2 to 64.1%) against a CMRS. At a fixed value of sensitivity near 95% (95.5% nearest upper value [CI, 87.6 to 98.5%]), the specificity was 25.6% (CI, 18.8 to 33.7%). In comparison, the laboratory-based CRP test at a fixed value of sensitivity near 90% (90.9% nearest upper value [CI, 81.6 to 95.8%]) would achieve a specificity of 69.0% (CI, 60.6 to 76.3%) (CRP value of 12.3 mg/liter) against the CMRS. Similarly, at a fixed value of sensitivity near 95% (95.5% nearest upper value [CI, 87.5 to 98.4%]), the specificity was 45.7% (CI, 37.4 to 54.3%) (CRP value of 6.2 mg/liter).

Considering the Xpert-MTB-HR-Prototype test as a stand-alone diagnostic test, the specificity should ideally be at least 95% against a CMRS. At a specificity of 95.3% (nearest value) (CI, 90.2 to 97.9%), the test achieves a sensitivity of 65.7% (CI, 53.7 to 75.9%). In comparison, the CRP test performance at a specificity of 95.4% (nearest value) (CI, 90.2 to 97.9%) resulted in a sensitivity of only 13.6% (CI, 7.3 to 23.4%) (CRP value of 253.6 mg/liter).

### Performance evaluation of Xpert-MTB-HR-Prototype against Xpert MTB/RIF.

**FIG 3:**
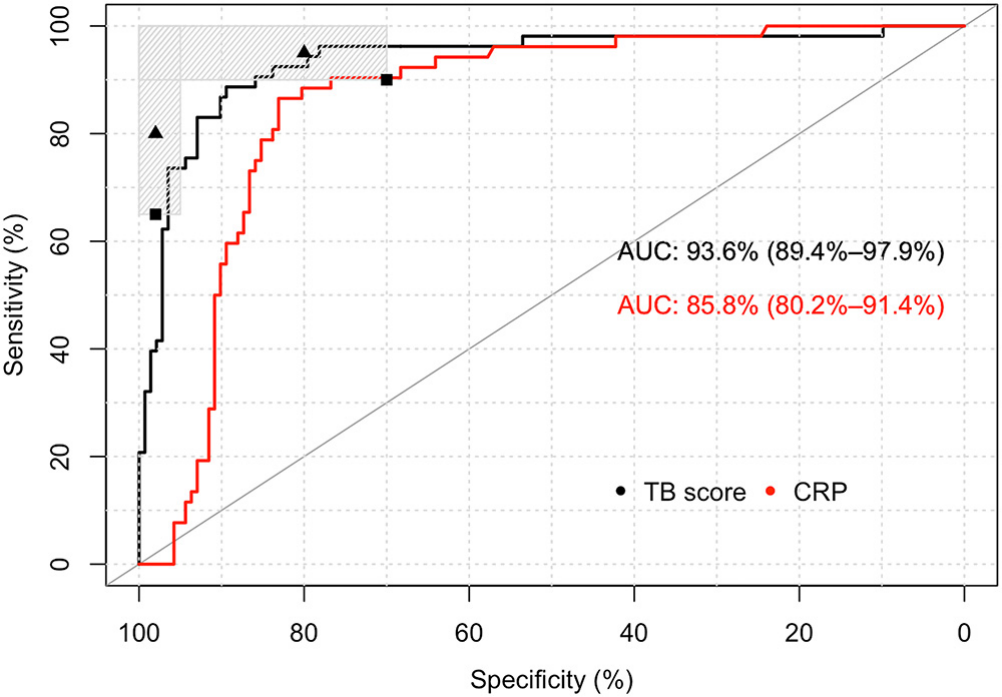
ROC curve against Xpert MTB/RIF on the first sputum sample as a reference standard for the Xpert-MTB-HR-Prototype test and the laboratory-based CRP test. The shaded regions represent areas with sensitivity and specificity combinations that meet at least the minimal target of one of the TPPs (triage or nonsputum diagnostic). The triangles represent the optimal targets, and the squares represent the minimal targets.

The TPP of the WHO specified a minimal sensitivity and specificity for a non-sputum-based triage test against Xpert MTB/RIF as the most likely confirmatory test in high-burden, resource-limited settings. Therefore, we assessed the performance against a single Xpert MTB/RIF assay on the first sputum sample. The AUC for Xpert-MTB-HR-Prototype with this reference standard was 0.94 (CI, 0.89 to 0.98), whereas the AUC for the CRP test was 0.86 (CI, 0.80 to 0.91) (Fig. 3). Using DeLong’s test, the AUROC for Xpert-MTB-HR-Prototype was significantly higher than that of the CRP test (*P* = 3.85e−3) (9). Optimized to achieve the highest Youden index, Xpert-MTB-HR-Prototype had an 88.7% sensitivity (CI, 77.4 to 94.7%) and an 89.4% specificity (CI, 83.3 to 93.5%) (Fig. 3). Considering the Xpert-MTB-HR-Prototype test as a triage test at a fixed value of sensitivity near 90% (90.6% nearest upper value [CI, 79.8 to 95.9%]), the Xpert-MTB-HR-Prototype specificity was 85.9% (CI, 79.3 to 90.7%). At a fixed value of sensitivity near 95% (96.2% nearest upper value [CI, 87.3 to 99.0%]), the specificity was 78.2% (CI, 70.7 to 84.2%).

In comparison, at a CRP test value of 60.3 mg/liter (optimized to achieve the highest Youden index against the Xpert MTB/RIF reference standard), the sensitivity was 86.5% (CI, 74.7 to 93.3%), and the specificity was 82.4% (CI, 75.3 to 87.8%). A fixed value of sensitivity near 90% (90.4% nearest upper value [CI, 79.4 to 95.9%]) achieved a specificity of 76.8% (CI, 69.2 to 82.3%) (CRP value of 29.7 mg/liter), and at a sensitivity near 95% (96.2% nearest upper value [CI, 87.0 to 98.4%]), the specificity was 57.0% (CI, 48.9 to 64.9%) (CRP value of 10.2 mg/liter) (Fig. 3).

When comparing Xpert-MTB-HR-Prototype and CRP scores for each sample, we observed that the samples misclassified by Xpert-MTB-HR-Prototype and CRP are largely the same (see Fig. S2, 4th quadrant, in the supplemental material).

Subgroup analyses of sensitivity and specificity at the optimal TB score cut point by CD4 counts and by the number of symptoms for Xpert-MTB-HR-Prototype are provided in Table 2 (ROC curves and box plots for subgroups are in Fig. S4 and S5). Subgroup analysis assessing specificity according to LTBI status as defined by IGRAs determined a lower specificity in patients with LTBI of 89.1% (CI, 77.0 to 95.3%) versus 94.0% (CI, 86.7 to 97.4%), but confidence intervals were overlapping (box plots by LTBI status in Fig. S6). In the participants identified to have subclinical TB, 0 out of 5 had a positive Xpert-MTB-HR-Prototype test when the optimized cut point was used.

**TABLE 2:** Xpert-MTB-HR-Prototype subgroup analyses at the optimal TB score cut point against Xpert MTB/RIF on the first sample as the reference standard^*a*^

Subgroup	No. of samples	% sensitivity (CI)	% specificity (CI)
CD4 count (cells/mm^3^)			
≥200	129	75.0 (50.5–89.9)	94.7 (88.9–97.5)
<200	56	93.8 (79.9–98.3)	66.7 (46.7–82.0)

No. of symptoms			
≤3	19	100 (70.1–100)	80.0 (49.0–94.3)
>3	176	86.4 (73.3–93.6)	90.2 (83.9–94.2)

LTBI status (in non-TB)			
Non-LTBI	82		93.9 (86.5–97.4)
LTBI	46		89.1 (77.0–95.3)

a Abbreviations: CI, confidence interval; LTBI, latent tuberculosis infection.

An exploratory analysis assessed the time to culture positivity against the TB score (Fig. S6). From this analysis, it was evident that many of those cases not captured by cut points of sensitivity set at 90% or 95% were those with paucibacillary disease, i.e., a long time to culture positivity, with many also not captured by Xpert MTB/RIF (Fig. S2).

Considering the implementation of Xpert-MTB-HR-Prototype as a triage test at a sensitivity of 90.6% (specificity at 85.9%) followed by Xpert MTB/RIF as a confirmatory test, 68 out of 196 patients (excluding 5 subclinical cases) would have a positive result with the triage test (48 true-positive and 20 false-positive cases) and would have to be confirmed by Xpert MTB/RIF on the first sputum sample. The algorithm of Xpert-MTB-HR-Prototype followed by Xpert MTB/RIF would miss 19 cases identified by the CMRS compared to 14 cases missed with a strategy of Xpert MTB/RIF alone on all presumed TB patients. This strategy would utilize 196 Xpert-MTB-HR-Prototype cartridges and 69 Xpert MTB/RIF cartridges. In comparison, the CRP test (at a sensitivity of 90.4% and a specificity of 76.8%) would miss the same number of cases but utilize 81 Xpert MTB/RIF cartridges in addition to the 196 CRP tests.

## DISCUSSION

This study assesses for the first time the performance of a novel, blood-based early-prototype test that is based on the quantification of mRNA levels of 3 host genes in well-characterized samples from PLHIV presenting with symptoms suggestive of TB in settings where the disease is highly endemic. The study establishes the feasibility of implementing the 3-gene signature in a well-proven cartridge-based testing system. Furthermore, the study confirms the performance of the signature in this cartridge system in comparison to various studies using RNA sequencing and quantitative PCR (qPCR) and again shows its ability to meet the minimal WHO TPP for a triage test (10). The signature that was implemented in the cartridge is the most extensively validated transcriptomic signature to date, including validation by an independent group in a large cohort of >1,400 patients (11). It was originally derived by meta-analysis of whole-blood gene expression data of 3 clinical cohorts from 6 different countries and subsequently validated with data sets from 11 countries, in which it distinguished active TB disease from other diseases, with sensitivity and specificity estimates of 81% and 74%, respectively (10). Importantly, the 3-gene signature was also shown to predict progression from LTBI to active TB up to 6 months prior and associated with a risk of subclinical active TB posttreatment in independent cohorts (12).

Our study shows that in HIV-positive patients with active TB, the performance of Xpert-MTB-HR-Prototype as a triage test is similar to that of a laboratory-based CRP assay at a preselected 90% sensitivity compared to a CMRS. However, the TPP for a non-sputum-based triage test by the WHO is specified against Xpert MTB/RIF as the most likely confirmatory test in high-burden, resource-limited settings. When using Xpert MTB/RIF as the confirmatory test on the first sputum sample in a triage scenario, the prototype cartridge was significantly better than the CRP test. Using Xpert MTB/RIF on the first sputum sample as a reference standard at a preselected sensitivity of 95% (i.e., the optimal target), Xpert-MTB-HR-Prototype achieved 78% specificity, which approaches the optimal TPP for a nonsputum triage test specified by the WHO. In contrast, at 95% sensitivity, the laboratory-based CRP assay has substantially lower specificity (57%).

This significantly higher accuracy of Xpert MTB-HR-Prototype than of the CRP assay has important implications for a triage test, especially in an HIV-negative patient population. In HIV-negative cohorts, the performance of the CRP test is highly likely to be more affected by non-TB inflammatory conditions. CRP reflects ongoing inflammation and/or tissue damage across a broad range of diseases, including infections, allergic complications of infection, autoimmune diseases, and malignancies such as lymphoma, carcinoma, and sarcoma. Examples of routine clinical uses of CRP include (i) assessment of disease activity in inflammatory conditions (e.g., rheumatoid arthritis, Crohn’s disease, psoriatic arthropathy, rheumatic fever, acute pancreatitis, and familial fevers), (ii) diagnosis and management of infections such as bacterial endocarditis and intercurrent infection in lupus and leukemia, and (iii) prediction of future atherothrombotic events, including coronary events, stroke, and progression of peripheral arterial disease (13). When used in an HIV-negative population for triage where other inflammatory conditions are more prevalent, the specificity of the CRP test will be further reduced and result in a higher number of false-positive results requiring additional confirmatory tests. In contrast, the performance of the 3-gene signature in other studies was shown to be lower in HIV-positive patients with TB than in HIV-negative patients, which might be related to the lesser immune stimulation in HIV-positive patients (5). Hence, Xpert-MTB-HR-Prototype can be expected to have higher accuracy in a larger cohort of those without HIV and may be able to achieve the optimal TPP for a nonsputum triage test.

At 90% sensitivity, compared to the CMRS, as expected, both the prototype and CRP tests missed 6 patients. The median (and average) CD4 count for the 6 patients missed in both tests is 524 cells/mm^3^. These results suggest that in PLHIV, some patients may not mount a sufficient/detectable host response as represented by either Xpert-MTB-HR-Prototype or the CRP test, even though they are not substantially immunosuppressed. The paucibacillary nature of the disease in these patients might be contributing (all but one of these patients were smear negative) to the limited immune stimulation. Collectively, our results suggest that the 10% of patients with active tuberculosis (ATB) missed by the prototype cartridge are those with a limited host response due to paucibacillary infection that may lead to a longer time to positivity.

Thus, a larger cohort including HIV-positive and HIV-negative patients should be explored to assess how the two tests perform in these subgroups in comparison. Also, the current CRP results were generated using a laboratory-based platform (Abbott Architect), while point-of-care CRP tests are available and most suitable for use as a triage test (4). Thus, a comparison of Xpert-MTB-HR-Prototype to a point-of-care CRP test would be a logical next step and an important study for the real-world application of both tests to assess whether the results observed here in comparison to the CRP test hold up when a simple lateral-flow assay for CRP is used.

The results of the Xpert-MTB-HR-Prototype test as a stand-alone diagnostic test seen in our study hold promise particularly for patients who are unable to provide a sputum sample, e.g., PLHIV and children. These data also need to be further validated, and studies to assess the early prototype for triage testing in children are ongoing. Considering the increased use of the Xpert MTB/RIF Ultra assay, future studies will need to include comparisons using Ultra to understand the role of Xpert-MTB-HR-Prototype in these settings. The study by Sweeney et al. also suggested that the 3-gene signature may be useful for monitoring the response to TB treatment as the TB score normalizes in patients with successful treatment, and studies to evaluate Xpert-MTB-HR-Prototype for treatment monitoring are also ongoing (5). Given that we tested an early prototype of the cartridge, further improvements in its diagnostic performance are conceivable with refinements in its development.

Although these data hold promise for the performance of Xpert-MTB-HR-Prototype, widespread access and use will depend on costs and programs that enable access to the cartridge. Widespread use is unlikely to occur if costs are much higher than those of currently available Xpert MTB/RIF tests. Current pricing systems for other Xpert products in countries with a high burden of tuberculosis are likely to be employed when this product is available to the market.

The study has several limitations. First, all samples used in the study were from HIV-positive subjects. In active screening, a large proportion of subjects are expected to be HIV negative, where the 3-gene TB signature has been shown to have a higher accuracy. Second, while this was a nested case-control study, the degree of bias is probably very limited, as controls were selected from the same population as the cases, and the characteristics of the population in terms of TB prevalence and smear status are similar to those of the total cohort. Third, as the study was used as a derivation cohort to select an optimal cut point and use case, the performance needs to be further evaluated in a validation cohort with a set cut point. Fourth, the study assessed samples from South Africa and Peru only, and the sample size from Peru was too small to perform subgroup analysis by country of origin. While South Africa contributes a significant share of the global TB/HIV epidemic, it will be critical to validate the host-marker-based signature in other countries where there are more coinfections likely (e.g., in equatorial Africa), other strains circulating, and other host genetic backgrounds. However, as we note above, the 3-gene TB signature itself has been extensively validated across different countries, thus substantially reducing the likelihood of effects of other circulating strains and host genetics on its performance. Additionally, we may have disadvantaged the prototype cartridge by excluding extrapulmonary TB since this may result in a host response similar to that in pulmonary TB. Thus, the prototype cartridge could have detected cases that otherwise would have been missed or required invasive sampling. However, further investigation is needed to confirm this claim. Finally, another limitation is that testing of the samples was performed nonblinded to the results of the reference standard by the test manufacturer. However, given that this was the first accuracy study of the cartridge, and no preset cut point was available, blinding was not possible as a cut point needed to be defined. Given that no subjective interpretation is necessary for the test, this is not likely to introduce bias. And importantly, all data were made available to FIND for analysis independently.

The study also has several strengths. The protocol and analysis plan were predefined. The assessment was done in a cohort that presented with symptoms suggestive of TB in a high-prevalence setting. The controls were chosen from the same cohort as the cases. The prevalence and smear status of the cohort are similar to what has been reported in other TB accuracy studies (14). The patients enrolled were extremely well characterized, with four sputum cultures on two independent samples, blood cultures, large-volume urine Xpert MTB/RIF, and 2-month follow-up to ensure the resolution of symptoms in the absence of treatment in patients who had a negative microbiological work-up. This extensive characterization allowed a very good estimate of the true TB status of patients, which is typically extremely difficult in PLHIV (15). Additional analyses are under way using Xpert-MTB-HR-Prototype on patients in diverse geographical locations to further evaluate its performance.

In summary, in this first accuracy study of a novel blood-based host marker assay, we show the possible value of the assay for diagnosis in a vulnerable and very difficult-to-diagnose population living with HIV.

## Supplementary Material

Supplemental file 1
