# Supplementary material for: Diagnostic Accuracy Study of a Novel Blood-Based Assay for Identification of Tuberculosis in People Living with HIV
Source: J Clin Microbiol. 2021 Feb 18;59(3):e01643-20. doi: 10.1128/JCM.01643-20 (PMC8106701; doi:10.1128/JCM.01643-20)
Supplement: Supplemental file 1 [file JCM.01643-20-s0001.pdf]

Supplementary Figures

Fig. S1. Specimen collection flow

Fig. S2. Correlation of CRP and TB-score of Xpert Prototype.

Fig. S3. ROC curve for subgroups analyses (A) By CD4 count and (B) for number of symptoms at optimal TB-score cut-point against Xpert on first sample as reference standard

Fig. S4. Box plots for subgroups analyses (A) By CD4 count and (B) for number of symptoms at optimal TB-score cut-point against Xpert on first sample as reference standard

Fig. S5. Box plots by LTBI status

Fig. S6. Time to positivity on culture versus TB-score of Xpert Prototype

Table S1. Diagnostic categories**.**

Table S2: Estimates of accuracy of the TB Host Response Prototype in subgroups at optimal threshold

[#] Disclaimer: Product in development. Not for use in diagnostic procedures. Not reviewed by any regulatory body.

Supplement Figure 1: Specimen collection flow

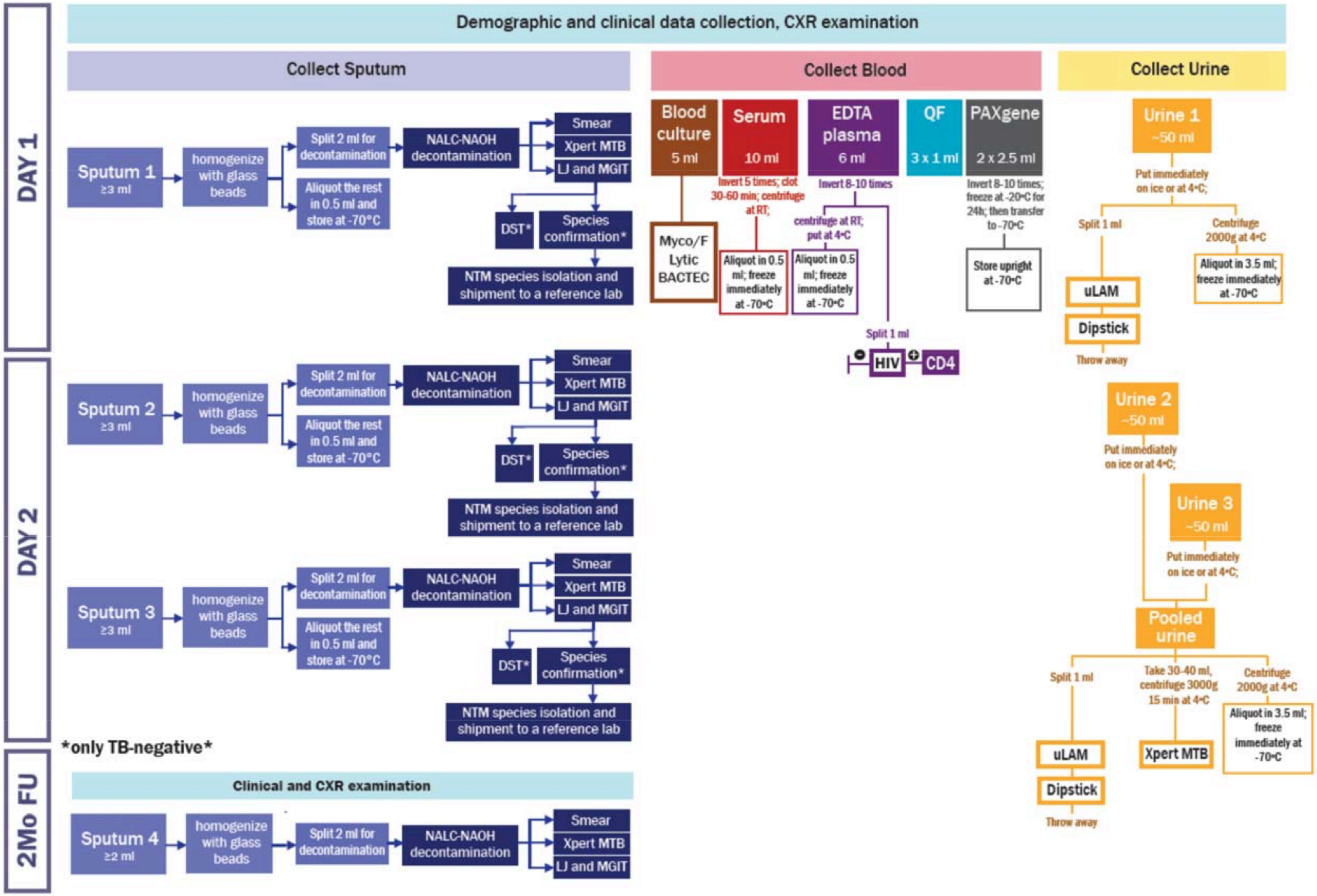

Disclaimer: Product in development. Not for use in diagnostic procedures. Not reviewed by any regulatory body.

Supplement Figure 2: Correlation of CRP and TB-score of Xpert Prototype

CRP on Y-axis in log10-scale. TB-score on X-axis. The vertical and horizontal green lines represent 90% sensitivity threshold values for TB-score and CRP (29.7 mg/L). In case of CRP, samples detected at the 90% sensitivity threshold values will be the ones above the horizontal green line; for TB score, those will be the ones on the left of the vertical line. Color indicates which of those samples will be positive by the Xpert on the first sputum. Dots in the left panel are colored based on CMRS results; right panel colored based on results from Xpert on first sputum.

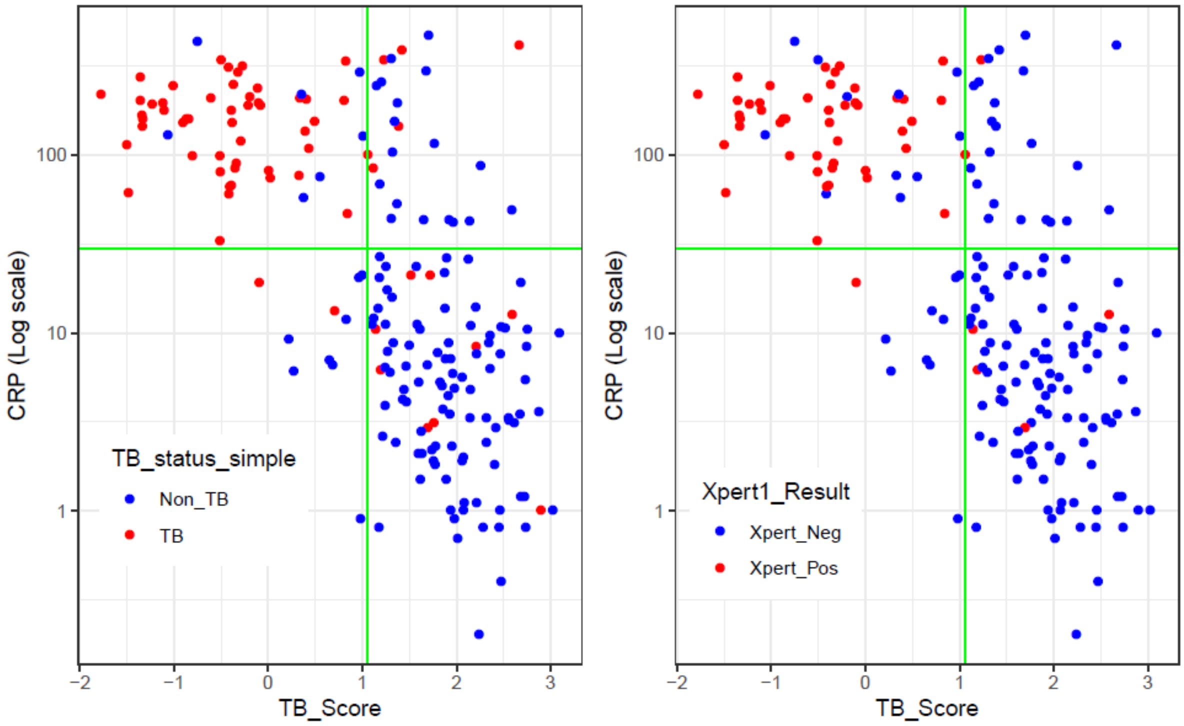

When comparing Xpert Prototype and CRP scores for each sample, we observed that the nine samples mis-classified by TB-score and CRP are largely the same (see 4th quadrant). Xpert on sputum also does not identify 5 out of these 9 patients identified by CMRS. Furthermore, this figure shows that when using Xpert alone on first sputum as a confirmatory test, the Xpert Prototype has a smaller number of false positives (20 FPs) compared to CRP (31 FPs), which increases the specificity of Xpert Prototype, and result in lower number of Xpert cartridges required as a confirmatory test compared to CRP.

Disclaimer: Product in development. Not for use in diagnostic procedures. Not reviewed by any regulatory body.

Supplement Figure 3: ROC curve for subgroups analyses (A) By CD4 count and (B) for number of symptoms at optimal TB-score cut-point against Xpert on first sample as reference standard.

(A)

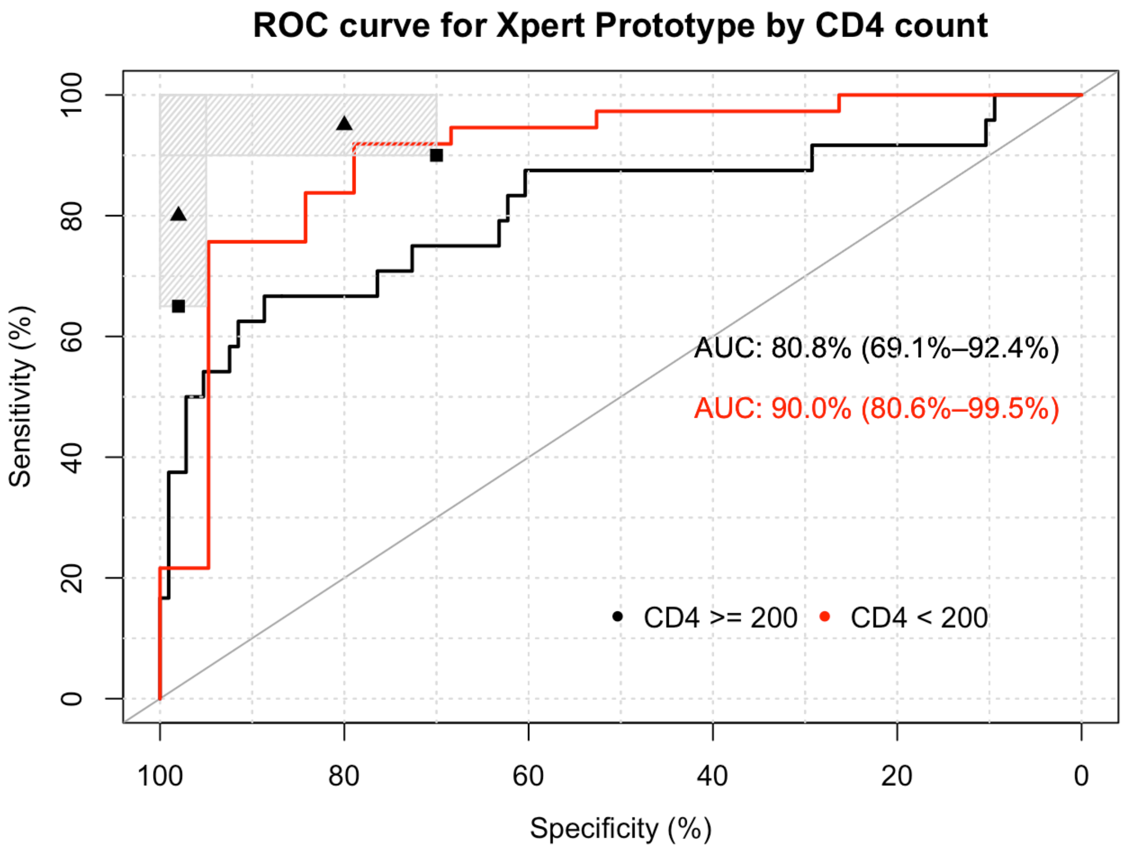

(B)

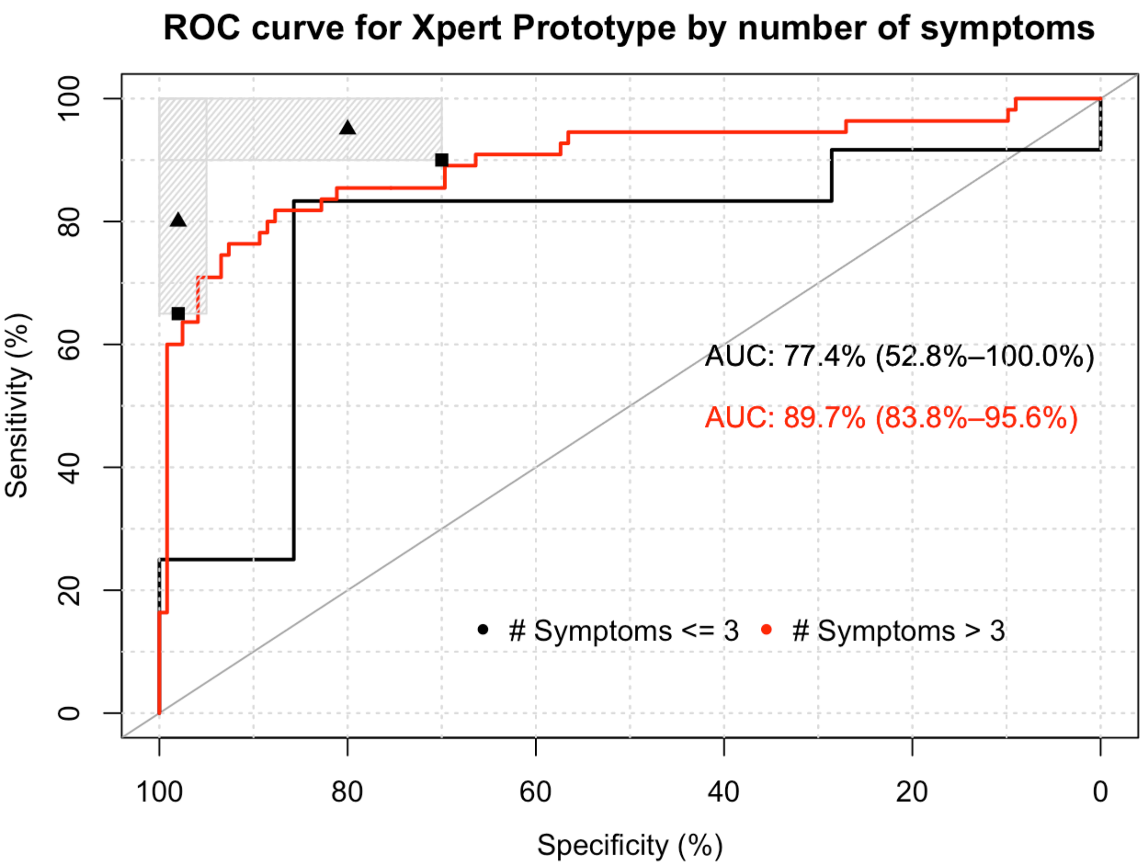
ROC curve for Xpert Prototype by number of symptoms
Sensitivity (%)
Specificity (%)
100
80
60
40
20
0
AUC: 77.4% (52.8%–100.0%)
AUC: 89.7% (83.8%–95.6%)
Symptoms <= 3
Symptoms > 3

Supplement Figure 4: Box plots for subgroups analyses (A) By CD4 count and (B) for number of symptoms at optimal TB-score cut-point against Xpert on first sample as reference standard

(A)

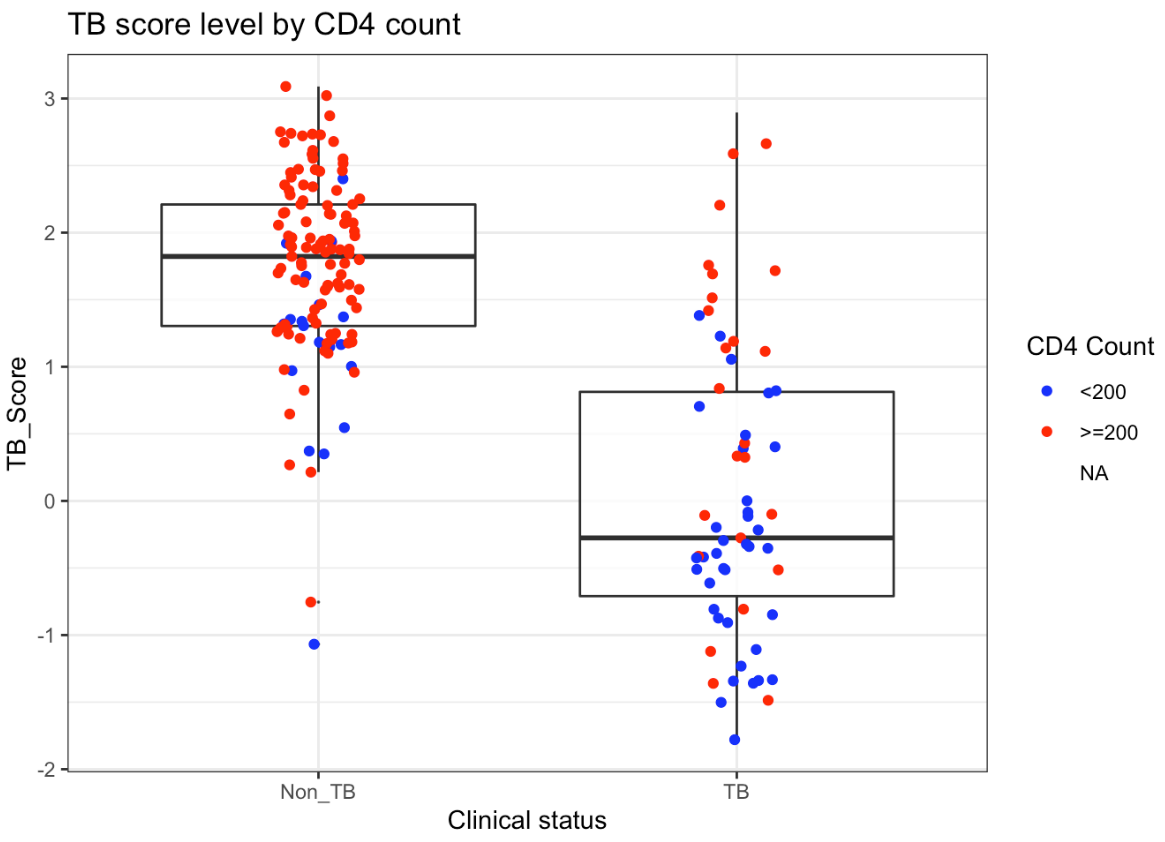

(B)

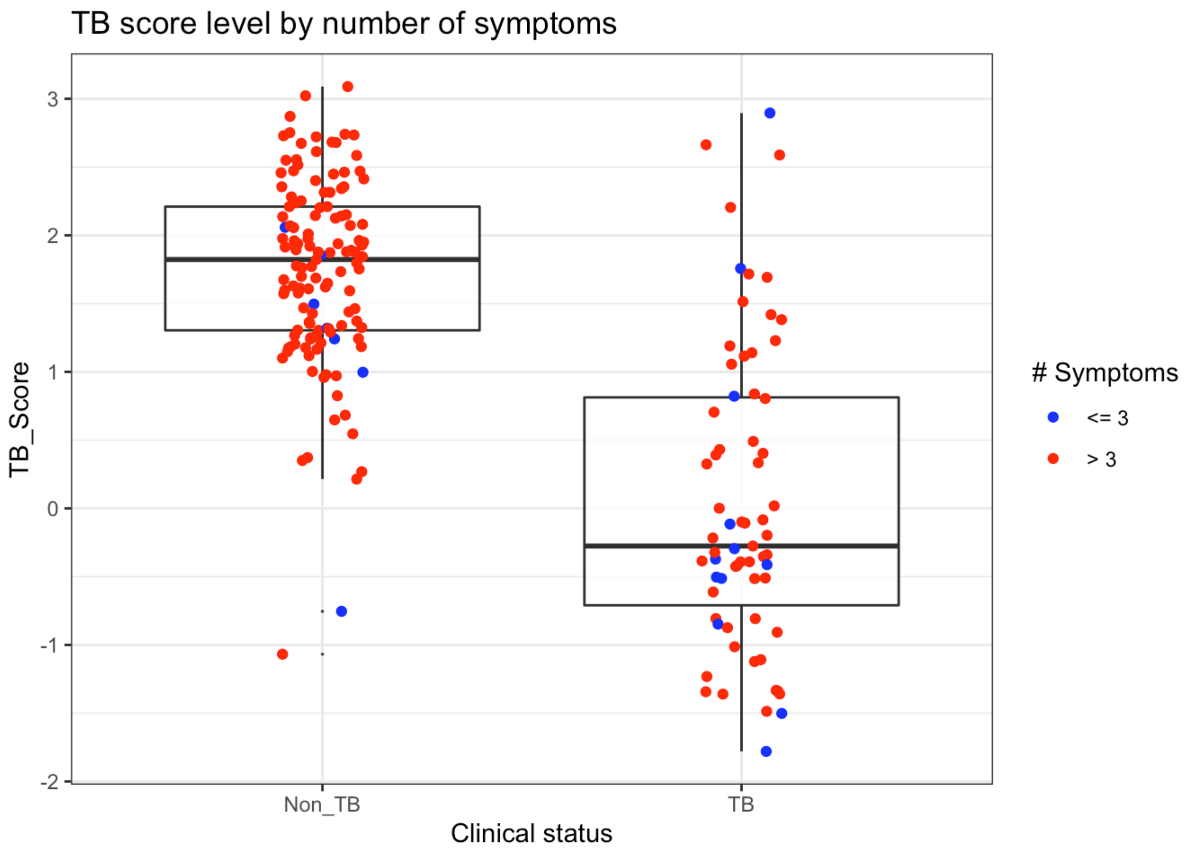

TB score level by number of symptoms
3
2
1
0
-1
-2
TB_Score
Non_TB
TB
Clinical status
Symptoms
<= 3
> 3

Supplement Figure 5: Box plots by LTBI status

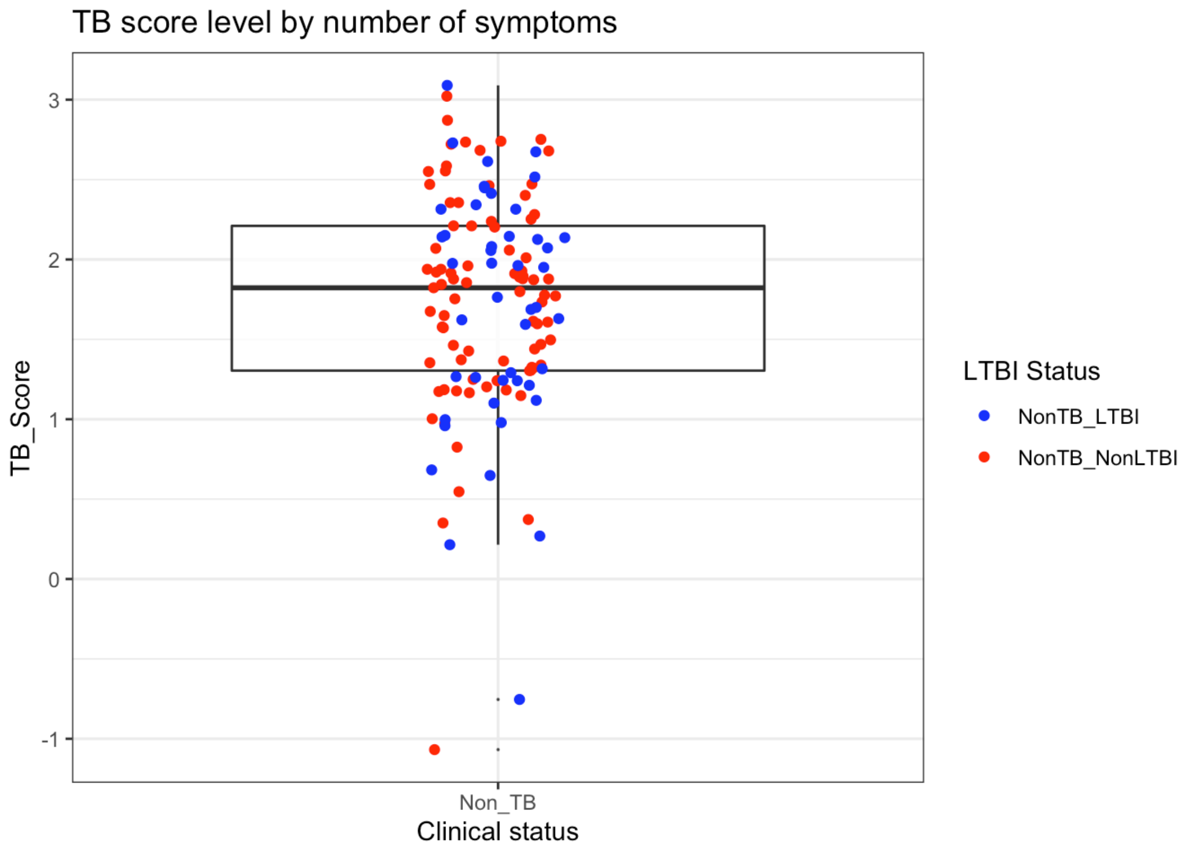

Supplement Figure 6: Time to positivity on culture versus TB-score of Xpert Prototype

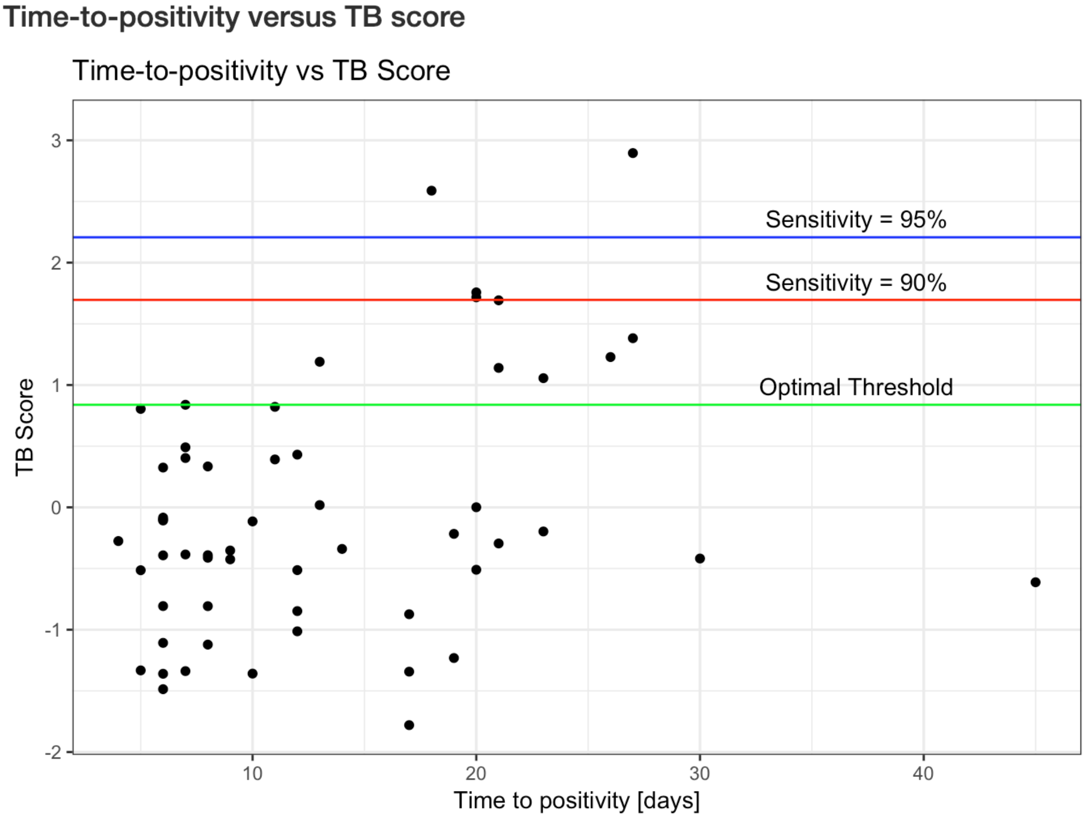

Caption: Green line at TB-score cut-point with an optimized AUC; Red line: TB-score cut-point set to the minimal sensitivity of a triage test at 90%; Blue line: TB-score cut-point set close to the optimal sensitivity of a triage test at 95%.

Supplement Table 1: Diagnostic categories

Category	
**Definite TB**	**Any culture or any Xpert (baseline) positive for MTB**<br>≥1 Positive culture (solid, liquid, sputum or blood) & confirmed MTB complex at baseline (Cross-contamination: A single LJ culture with ≤ 20 colonies or a single MGIT culture with MTB growth ≥28 days per patient are excluded from analysis)<br>Or<br>≥1 Positive Xpert (sputum or urine) at baseline
**Possible TB**	**Any patient not meeting definite TB or Not TB classification who is started on TB treatment**<br>Empiric TB treatment started by the healthcare provider
**Not TB**	**All microscopy, culture and Xpert tests negative for MTB, not started on TB treatment, recovers and has negative follow-up tests**<br>All culture negative (sputum, blood, incl. follow-up, with at least 2 LJ or MGIT with no culture growth after >56 days and >42 days)<br>AND<br>At least 2 valid negative culture or Xpert results from 2 or more independent samples obtained from 2 or more different anatomic sites, such as blood, sputum, or urine<br>AND<br>All Xpert negative (incl. follow-up)<br>AND

	All smear microscopy results negative (incl. follow-up)<br>AND<br>Treatment not initiated by healthcare providers<br>AND<br>Improvement or full recovery at 8-week follow-up in the absence of TB treatment
**Subclinical**	**Patients with negative tests for MTB at baseline and positive tests in follow-up**<br>Positive sputum culture and/or sputum Xpert and/or sputum smear on follow-up
**Unclassifiable**	**All participants that do not fall into groups "Definite TB", "Not TB" or "Possible TB"**<br>i.e.:<br>No symptom resolution on follow-up (same or worse) for baseline negative participants<br>Or<br>Loss to follow-up for baseline negative participants<br>Or<br>Passed away for baseline negative participants<br>Or<br>Insufficient laboratory results (i.e. participants with <2 valid cultures)<br>OR<br>Baseline smear microscopy positive but culture and Xpert negative

Supplementary Table 2: Estimates of accuracy of the TB Host Response Prototype in subgroups at optimal threshold*

Subgroup	Estimate	Value	LCI	UCI	Reference
Smear Neg Culture Pos	Sensitivity	0.5217	0.3296	0.7076	Culture
Smear Neg Culture Pos	Specificity	NA	0.8632	0.9574	Culture
Smear Neg Culture Pos	Sensitivity	0.8	0.4902	0.9433	Xpert1
Smear Neg Culture Pos	Specificity	0.6923	0.4237	0.8732	Xpert1
Smear Neg Culture Pos	Sensitivity	0.6429	0.3876	0.8366	Xpert
Smear Neg Culture Pos	Specificity	0.6667	0.3542	0.8794	Xpert
Smear Pos Culture Pos	Sensitivity	0.9091	0.7884	0.9641	Culture
Smear Pos Culture Pos	Sensitivity	0.907	0.784	0.9632	Xpert1
Smear Pos Culture Pos	Sensitivity	0.907	0.784	0.9632	Xpert

***Specificity is not reported for those categories in which the samples were not large enough**
